# Exploring Serum Neutrophil Gelatinase-associated Lipocalin as a Potential Biomarker in Obsessive-Compulsive Disorder

**DOI:** 10.1192/j.eurpsy.2026.11009

**Published:** 2026-07-31

**Authors:** C. Selvi, I. Ekmekci Ertek, Ö. Gülbahar

**Affiliations:** 1 Department of Psychiatry; 2 Department of Medical Biochemistry, Gazi University Hospital, Ankara, Türkiye

## Abstract

**Introduction:**

Obsessive-compulsive disorder (OCD) is a chronic psychiatric disorder affecting approximately 2% of the population, characterized by intrusive thoughts and repetitive behaviors that impair functioning. Although its pathogenesis remains incompletely understood, accumulating evidence implicates inflammatory mechanisms in its development. Neutrophil gelatinase-associated lipocalin (NGAL) is a pro-inflammatory glycoprotein primarily secreted by glial cells, involved in regulating neuroinflammatory responses and neuronal injury. Only a limited number of studies have assessed NGAL in OCD; the first reported elevated serum levels in patients compared with controls, with no association to symptom severity.

**Objectives:**

This study aimed to compare serum NGAL levels in drug-naïve OCD patients and healthy controls, and to examine possible associations with sociodemographic variables and clinical severity.

**Methods:**

In this case-control study, 29 drug-naïve patients diagnosed with OCD according to DSM-5 criteria and 28 age- and sex-matched healthy controls were included. Clinical severity was assessed using the Yale-Brown Obsessive-Compulsive Scale (Y-BOCS). Venous blood samples were collected from all participants, and serum NGAL concentrations were measured using an enzyme-linked immunosorbent assay (ELISA). Sociodemographic data were recorded through structured interviews. Statistical significance was assessed using Mann-Whitney U test, T-test and Pearson/Spearman correlation for relationships between variables.

**Results:**

Serum NGAL levels were analyzed in OCD patients (n=29) and healthy controls (n=28). Median NGAL concentration was (103.81 (85.17-153.99)**ng/mL)** in the OCD group and (140.13 (92.61-358.72)ng/mL) in controls, with no statistically significant difference (p=0.089).Sex-stratified analyses also revealed no significant differences between groups. No correlation was found between NGAL levels and severity of symptoms among OCD patients. (Total Y-BOCS score rₛ = 0.041; compulsions rₛ = 0.036; obsessions rₛ = 0.075, (p >0.05))

**Conclusions:**

Serum LCN2 levels did not differ between drug-naïve OCD patients and healthy controls, and no association with symptom severity was observed. Conflicting findings in the literature suggest that LCN2 alone is unlikely to serve as a reliable biomarker for OCD. The absence of significant differences may reflect sample size limitations or the complex role of LCN2 in neuroinflammation, possibly consistent with the inverted U-shaped effect described in other psychiatric disorders. Comparable smoking status and BMI between groups reduce the likelihood of major confounding. Larger longitudinal studies are warranted to clarify the role of LCN2 in the neurobiology of OCD

**Disclosure of Interest:**

C. Selvi Grant / Research support from: SCIENTIFIC RESEARCH PROJECTS COORDINATION UNIT (BAP) / GAZI UNIVERSITY, I. Ekmekci Ertek: None Declared, Ö. Gülbahar: None Declared

